# 

**Published:** 1891-11

**Authors:** 


VOL. XII.] *PUBLISHED MONTHLY AT THREE DOLLARS A YEAR. SINGLE COPIES, THIRTY CENTS.* [NO. 11.

Entered at the Post Office, February 3rd, 1891, at New York as Second-class Matter.
Copyright, 1891, by W. A. Conklin, and R. S. Huidekoper.

### THE JOURNAL OF COMPARATIVE MEDICINE AND VETERINARY ARCHIVES.

EDITED BY

W. A. CONKLIN, Ph. D., D. V. S.,

*Director of Zoological Gardens, New York.*

RUSH SHIPPEN HUIDEKOPER, M. D., Veterinarian, (*Alfort.*)

*Professor of Sanitary Medicine and Veterinary Jurisprudence, American Veterinary College, New York.*

NOVEMBER, 1891.

All communications and payments should be addressed to

**MANAGING EDITOR JOURNAL COMP. MED. AND VET. ARCH.**

FORTY-SECOND STREET AND PARK AVENUE, NEW YORK.

Printed for the Editors by

WILLIAM R. JENKINS, PUBLISHER,

NEW YORK.

**LONDON: BALLIERE, TINDALL & COX.**

